# SLAM Algorithm for Mobile Robots Based on Improved LVI-SAM in Complex Environments

**DOI:** 10.3390/s24227214

**Published:** 2024-11-11

**Authors:** Wenfeng Wang, Haiyuan Li, Haiming Yu, Qiuju Xie, Jie Dong, Xiaofei Sun, Honggui Liu, Congcong Sun, Bin Li, Fang Zheng

**Affiliations:** 1 College of Electrical Engineering and Information, Northeast Agricultural University, Harbin 150030, China; z11650@neau.edu.cn (W.W.);; 2 Key Laboratory of Equipment and Informatization in Environment Controlled Agriculture, Ministry of Agriculture and Rural Affairs, Hangzhou 310058, China; 3 Key Laboratory of Smart Farming Technology for Agricultural Animals, Ministry of Agriculture and Rural Affairs, Wuhan 430070, China; 4 Engineering Research Center of Pig Intelligent Breeding and Farming in Northern Cold Region, Ministry of Education, Harbin 150030, China; 5 College of Animal Science and Technology, Northeast Agricultural University, Harbin 150030, China; 6 Agricultural Biosystems Engineering Group, Wageningen University, 6700 AA Wageningen, The Netherlands; 7 Intelligent Equipment Research Center, Beijing Academy of Agriculture and Forestry Sciences, Beijing 100097, China; 8 College of Informatics, Huazhong Agricultural University, Wuhan 430070, China

**Keywords:** multi-sensor fusion, SLAM, feature extraction, loop-closure detection, navigation

## Abstract

The foundation of robot autonomous movement is to quickly grasp the position and surroundings of the robot, which SLAM technology provides important support for. Due to the complex and dynamic environments, single-sensor SLAM methods often have the problem of degeneracy. In this paper, a multi-sensor fusion SLAM method based on the LVI-SAM framework was proposed. First of all, the state-of-the-art feature detection algorithm SuperPoint is used to extract the feature points from a visual-inertial system, enhancing the detection ability of feature points in complex scenarios. In addition, to improve the performance of loop-closure detection in complex scenarios, scan context is used to optimize the loop-closure detection. Ultimately, the experiment results show that the RMSE of the trajectory under the 05 sequence from the KITTI dataset and the Street07 sequence from the M2DGR dataset are reduced by 12% and 11%, respectively, compared to LVI-SAM. In simulated complex environments of animal farms, the error of this method at the starting and ending points of the trajectory is less than that of LVI-SAM, as well. All these experimental comparison results prove that the method proposed in this paper can achieve higher precision and robustness performance in localization and mapping within complex environments of animal farms.

## 1. Introduction

One important fundamental capability of mobile robots is autonomous navigation, which requires mapping and state estimation. Simultaneous localization and mapping (SLAM), as one of the state-of-the-art technologies, could provide a solution for real-time autonomous robot navigation by utilizing a variety of sensors. SLAM is a key technology that enables mobile robots to recover the poses and construct a complete map in unknown environments [1,2]. Based on the spatiotemporal relationships of the obtained observational data, robots can calculate the real-time position and attitude of the system, thus achieving localization and mapping [3]. However, due to the fact that most of the working environment of the robots is relatively complex with unexpected situations (e.g., sensor failures, data dropout, drastically-changing light, etc.), a single-sensor SLAM system may not be able to recover or re-localize quickly with poor environmental adaptability and insufficient robustness. Multi-sensor fusion is a technology that fully utilizes the advantages of different sensors, compensates for the disadvantages of single sensor, and improves adaptability and system performance in complex environments [4,5,6]. Table 1 shows the applicability of major existing sensors in SLAM, offering viable fusion schemes for multi-sensor fusion SLAM.

In the development history of SLAM technology, there are many excellent SLAM algorithms, which can be divided into lidar SLAM, visual SLAM, and multi-sensor fusion SLAM, according to the type of sensors SLAM uses [7].

In lidar SLAM, LOAM [8] is one of the classic algorithms, proposed by Zhang et al., which divides the SLAM problem into two parts and uses two algorithms to process in parallel. LOAM can achieve real-time construction of low-drift maps and compensates for lidar motion distortion, reducing the amount of point cloud registration through feature extraction. When computational resources are limited, obtaining low-drift motion estimation is challenging, leading to a decrease in performance. Shan et al. proposed LeGO-LOAM [9], which is derived from the LOAM framework and designed to run on lightweight embedded systems. Compared to LOAM, it has added lightweight and ground optimization. After segmenting the point cloud, it extracts planar and edge features and uses a two-step Levenberg–Marquardt (L-M) optimization method to solve the pose, building a more comprehensive map. LOAM and LeGO-LOAM utilize a single sensor (lidar) for SLAM mapping. Although researchers have integrated the inertial measurement unit (IMU) into lidar SLAM, in a loosely coupled manner, to assist in removing motion distortion, drift still occurs during large-scale testing [10]. To perform real-time state estimation and mapping in complex environments, Shan et al. extended the LeGO-LOAM framework to LIO-SAM [11], which is a tightly coupled lidar-inertial odometry framework. LIO-SAM replaces the frame-to-global map matching used in LOAM with frame-to-local map matching, improving significantly the system’s real-time capabilities.

In visual SLAM, the landmark algorithm PTAM [12] was proposed by Klein et al. in 2007. This algorithm separated tracking and mapping into two threads for the first time, introduced the concept of keyframe, and innovatively used nonlinear optimization in the SLAM back-end, which revolutionized scholars’ understanding of back-end optimization. However, there are limitations to PATM, such as restricted scene, complex initialization, and insufficient scalability. To improve these limitations, Mur-Artal et al. further refined the system architecture based on PTAM and proposed ORB-SLAM [13]. ORB-SLAM divides the mapping process into two threads: local mapping and loop detection, which uses ORB features, the scale and rotation invariant, as its core features [14]. ORB-SLAM can automatically initialize and operate in a variety of scenarios, demonstrating robustness to vigorous motion. PTAM and ORB-SLAM are applicable for SLAM mapping using a single sensor (monocular camera). In scenarios with fast motion and low-quality images, the mapping quality is degraded [15]. To enhance the overall performance of SLAM systems and ensure stable operation in complex environments, scholars have proposed VINS-MONO [16] and ORB-SLAM3 [17]. VINS-MONO and ORB-SLAM3 introduce a tightly coupled visual-inertial odometry (VIO) system, where IMU can obtain the pose of fast-moving objects, and the camera can continuously correct the cumulative error of IMU. VINS-MONO and ORB-SLAM3 can realize high-precision real-time localization and mapping in complex environments, with higher accuracy than that of the purely visual methods.

The LIO method can capture environmental details even under long-range conditions, but it is prone to show degradation in the unstructured scenarios [5,18]. The VIO method can achieve good results in environments with rich textures, but it is sensitive to light changes and initialization. To adapt to complex and variable environments and to enhance robustness and accuracy, Shan et al. proposed the LVI-SAM [19,20] framework by combining LIO-SAM with VINS-MONO. The framework consists of two parts: LIS and VIS, which achieves tight coupling between lidar, vision, and IMU through factor graphs, completing the fusion of various sensor data. The LVI-SAM framework fully utilizes the respective advantages of LIS and VIS.

However, in some complex scenarios, such as the working environment of livestock inspection robots, there are various adverse factors such as light changes, uneven road surfaces, and variations in speed. These factors will degrade the robot’s localization and mapping effectiveness, which will inevitably impact its navigation and inspection processes. In this paper, an advanced SLAM system that fuses lidar, camera, and IMU data based on LVI-SAM was proposed. This algorithm achieves good accuracy and robustness in localization and mapping in simulated complex environments of an animal farm. The main contributions of our work can be summarized as follows:

(1) The feature point detection method of VIS was improved to adding the SuperPoint feature point detection module, so that the system captures a certain number of feature points even in a harsh environment. The module combined with a Lucas–Kanade (LK) optical flow completes feature tracking better, improving the robustness and accuracy of feature tracking.
(2) The loop-closure detection method of LVI-SAM was improved to adding the scan context module, which performs dimensionality reduction on 3D point clouds, enhancing data processing and matching capabilities. VIS prior information is used to reduce the complexity of the matching process, improving the accuracy of loop detection and relocalization.
(3) The livestock inspection robot platform has been established, which has collected trajectory data from challenging scenarios in simulated complex environments of an animal farm, such as uneven road surfaces, light changes, and variations in speed. In the simulated environment, the robustness of the proposed algorithm is further verified.

## 2. Materials and Methods

### 2.1. Experimental Platform and Environment

**Figure 1:**
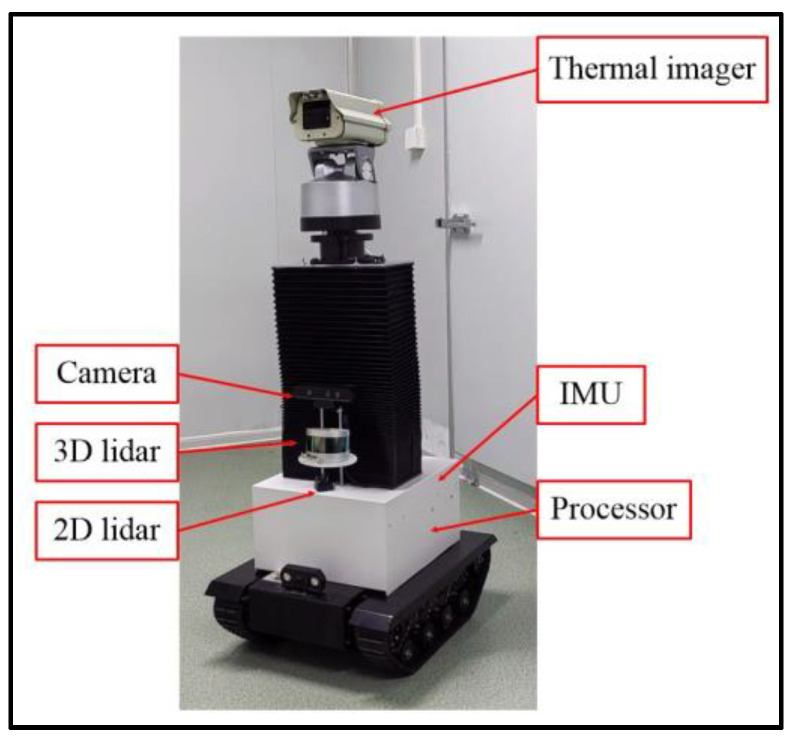
Experimental platform for livestock inspection robot.

An experimental platform for a livestock inspection robot [21,22,23] was set up as shown in Figure 1. The platform is equipped with various sensors, including 3D lidar, cameras, IMU, etc. The detailed parameters of sensors are shown in Table 2. The real-world scenario dataset in this paper was collected through this platform. The dataset is used to further evaluate the effectiveness of improving the LVI-SAM algorithm. Our algorithm and LVI-SAM run on a notebook with an Intel i7-13700H CPU (Intel, Santa Clara, CA, USA), 16 GB of RAM (Kingston, Foster, CA, USA), and an RTX 4060 GPU (NVIDIA, Santa Clara, CA, USA), using ROS-Noetic (Open Source Community) as the robot operating system.

### 2.2. Datasets Collection and Description

To verify the effectiveness of the algorithm proposed in this paper, experiments were conducted on the datasets of KITTI [24,25], M2DGR [26], and the real-world scenario. KITTI and M2DGR are public datasets, while the real-world scenario dataset was collected through the establishment of an experimental platform for a livestock inspection robot (Figure 1).

**Figure 2:**
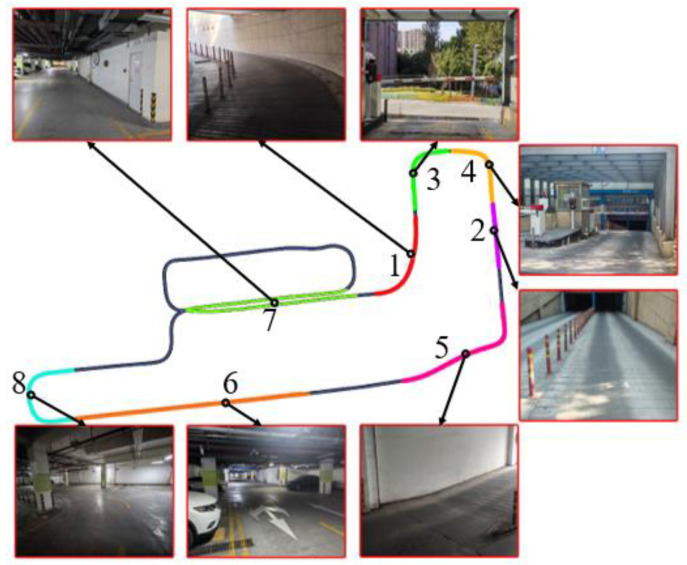
Experimental conditions and scenarios (the numbers correspond to Table 3).

(1) The KITTI dataset provides various types of data including images, lidar point clouds, and GPS/IMU, making it very suitable for researching the application of multi-sensor fusion technology in SLAM. This experiment selects KITTI sequence 05, which includes image data, lidar point cloud data, and GPS/IMU data collected in specific scenarios. It provides data from various complex environments, such as urban streets including large-angle turns and varying speeds. This sequence better validates the effectiveness and robustness of the improved LVI-SAM proposed in this paper.

(2) The M2DGR dataset is a new large-scale dataset for multi-sensor fusion SLAM. The dataset has a rich suite of sensors, encompassing a multitude of challenging scenarios, which is of significant importance for training and evaluating multi-sensor fusion SLAM algorithms. This experiment uses the sequence Street07 to verify the effectiveness of the method proposed in this paper. The route of this sequence is winding and tortuous, with a total length of 1104.07 m. The scenarios can easily cause sensor degradation, such as rapid turns, narrow areas (with similar characteristics resembling long corridor scenarios), and changes in lighting at night. This sequence is very challenging for both visual SLAM and lidar SLAM, and some existing methods do not perform very well on this sequence. 

(3) To better apply the method described in this paper to mapping and navigation for livestock inspection robots, special scenarios are combined with underground parking to simulate the complex environments of animal farms. The real-world dataset was collected through the experimental platform of livestock inspection robots, providing support for the next step of operation conditions for livestock inspection robots. To consider as many operational conditions as possible for a livestock inspection robot, the motion paths of this experimental platform have set up eight special scenarios to simulate the complex environments of animal farms, mainly including uphill, downhill, entering/exiting garage, undulating jolting, smooth straight movement, overlapping paths, and sharp turns at large angles, as shown in Table 3 and Figure 2. During the experimental process, some degradation scenarios were also set up, such as white walls, indistinct features, and changes in lighting. The starting and ending positions of the experimental platform were the same (making it easier to judge localization accuracy), and the total distance traveled was 663.7 m.

### 2.3. System Architecture

**Figure 3:**
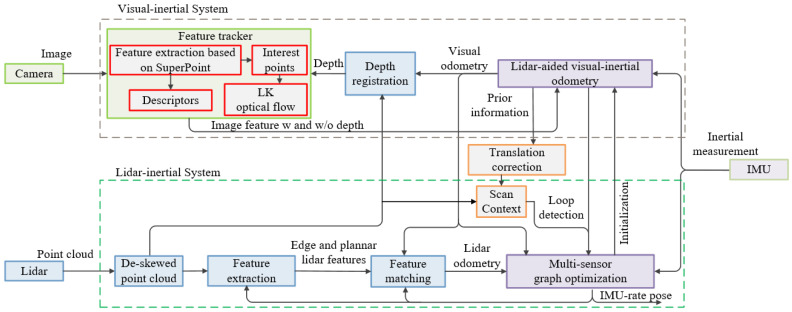
The system architecture of our method. The red solid-line boxes (SuperPoint) and the orange solid-line boxes (scan context) are the innovative parts of our method compared with LVI-SAM.

The proposed method is based on the modification and improvement of LVI-SAM. Figure 3 shows the schematic diagram of our SLAM algorithm. We have made some modifications to the system architecture of the original LVI-SAM algorithm, adding our innovative parts. The red solid-line boxes and the orange solid-line boxes, which represent the SuperPoint feature extraction module and the scan context loop detection module added to LVI-SAM, respectively, are the innovative parts of our method compared with LVI-SAM. The other colored boxes represent the modules of the original LVI-SAM framework. 

We have creatively applied the SuperPoint + LK optical flow method to monocular camera data for feature point detection and tracking in the VIS (brown dashed box), replacing the original Shi–Tomasi algorithm and achieving better results. We have added the scan context module to the loop-closure detection module of the original LVI-SAM, improving the system’s loop-closure detection method and enhancing the accuracy of the system’s loop-closure detection. 

Finally, the IMU pre-integration factor, visual odometer factor, lidar odometer factor, and loop-closure factor were jointly input into the factor graph (iSAM2) to solve the optimization problem for maximizing a posteriori estimation and completing the SLAM estimation problem.

### 2.4. Improved the Visual Feature Extraction Module

The original LVI-SAM used the popular Shi–Tomasi [27] corner detection algorithm to extract feature points from images. The Shi–Tomasi algorithm was improved based on the Harris algorithm, in order to detect corners by calculating the eigenvalues of the matrix with high computational efficiency, simple implementation, and good performance in various scenarios. 

However, in areas with sparse image textures, the Shi–Tomasi algorithm may possibly not be able to detect enough feature points. Therefore, our method proposed in this paper uses the SuperPoint algorithm to replace the Shi–Tomasi algorithm for feature point extraction to improve the visual feature extraction module of LVI-SAM, as shown in the red solid-line frame in Figure 3.

**Figure 4:**
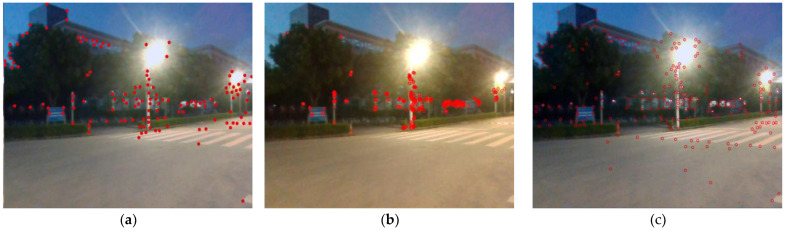
Comparison of Shi–Tomasi, ORB, and SuperPoint feature detection. (**a**) Shi–Tomasi algorithm. (**b**) ORB algorithm. (**c**) SuperPoint algorithm. The red circles indicate the feature points extracted by this method.

SuperPoint [28] is a feature point detection algorithm based on a deep learning network and a self-supervised deep learning model. This method can detect feature points under various visual conditions such as light changes, perspective changes, blurring, etc., showing higher robustness and accuracy in complex scenes. Therefore, we added the SuperPoint module to the original LVI-SAM framework. After incorporating the SuperPoint module, our algorithm can extract better visual feature points in some complex and harsh environments, providing a solid foundation for localization and mapping in the later stages of SLAM. Figure 4 shows images taken under insufficient nighttime lighting conditions. We use the Shi–Tomasi, ORB, and SuperPoint algorithms to detect feature points. SuperPoint captures 235 feature points, and ORB captures 213 feature points, while Shi–Tomasi only captures 135 feature points. As shown in Figure 4, ORB captures feature points mainly focused on the bright parts of the image, and for some areas that are blurry and insufficient in light, fewer feature points are captured. SuperPoint performs significantly better, capturing more abundant corners and adapting to more complex scenes with higher robustness. To enhance the system’s real-time performance, the visual front-end of our SLAM algorithm does not solely rely on the feature map of the SuperPoint descriptor for inter-frame feature matching. Instead, it combines SuperPoint feature points with the L-K optical flow method to achieve feature tracking.

### 2.5. Improved the Loop Detection Module

Loop detection is an indispensable part of SLAM systems. During the robot’s movement, loop detection is able to recognize and confirm returning to a previously visited location or area, which plays a crucial role in eliminating accumulated errors and improving localization and mapping accuracy. In the LVI-SAM system, loop-closure detection is jointly accomplished by VIS and LIS. Loop-closure constraints on relative pose are obtained through ICP matching, completing the entire loop-closure process. ICP matching is quite strict with the initial pose, and the loop path should not be too long [29]. Sometimes, the loop path is relatively long, which will decrease the accuracy and robustness of the aforementioned loop detection algorithm. Therefore, based on the original LVI-SAM, this paper integrates the scan context module into the LIS, performs scan context transformation on single-frame point clouds, confirms loop-closure frames, and thus enhances the accuracy and robustness of loop-closure detection, as shown in the orange solid-line frame in Figure 3.

**Figure 5:**
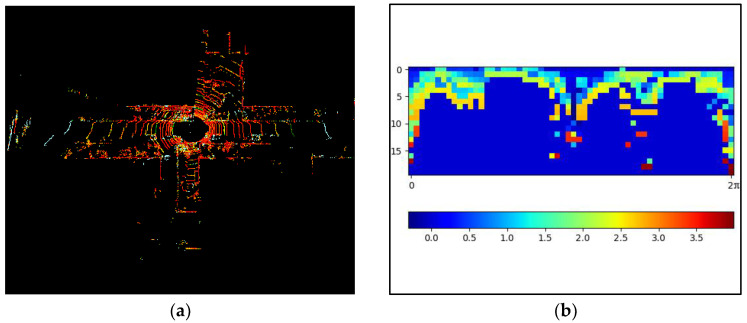
Frame 265 of the KITTI sequence 05 scan context transformation. (**a**) 3D point cloud. (**b**) Scan context.

Scan context [30] is a global localization method based on structural information, which can directly record 3D structural information and provides an efficient way to describe and compare the 3D structure of the surrounding environment. Based on the ideas of Kim et al., this algorithm reduces the dimensionality of single-frame 3D point cloud data by converting from Cartesian coordinates to polar coordinates and divides the entire scanning range (usually 360 degrees) into multiple equally wide sector regions (called bins). Each sector region was sequentially into a two-dimensional ring-sector matrix corresponding to the matrix rows and columns. The matrix element values are the maximum z-coordinate values of all points within the sector. The matrix can be expressed as:

(1) $$\emptyset \left(P_{ij}\right)=\max z\left(p\right)\;\;\;\;\;p\in P_{ij}$$

where $z\left(\;\right)$ is defined as a function through which the z-coordinate value of a point p is obtained, and $P_{ij}$ is defined as the set of all points in the sector corresponding to the i row and j column of the matrix. Figure 5 shows frame 265 of the KITTI sequence 05 scan context transformation. 

**Figure 6:**
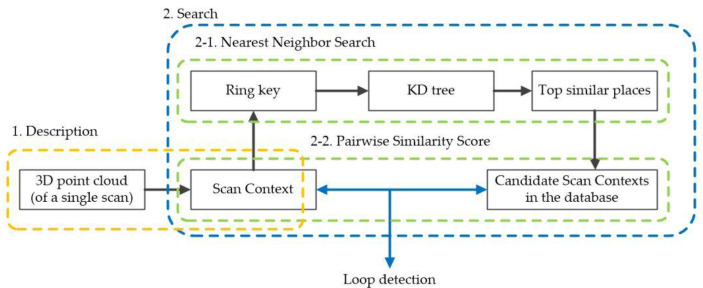
Scan context algorithm overview [30]. Copyright © 2018, IEEE.

**Figure 7:**
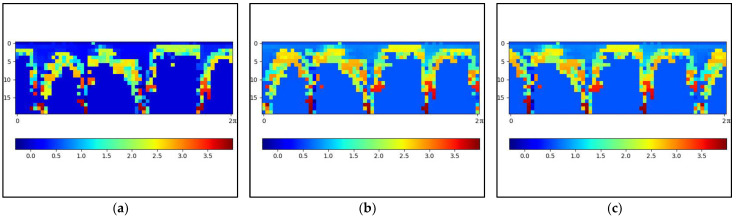
Frames 61 and 1105 of the KITTI sequence 05 scan context transformation. (**a**) The frame 61 scan context. (**b**) The frame 1105 scan context. (**c**) The Scan Context after translation of frame 1105.

Our algorithm introduces the scan context module into the LIS and completes the loop detection according to the process in Figure 6. Scan context effectively compresses data while preserving the structural information of the scene, but it makes the data sensitive to rotation at the same time. The scan context results of frames 61 and 1105 of the KITTI sequence 05 are shown in Figure 7a,b. Frames 61 and 1105 are 3D point cloud data captured at the same location but with different rotation angles. It can be seen that the scan context outlines of the two frames are basically the same, except that the order in the column direction is different, resulting in a translation. This will cause erroneous matching during the matching process, resulting in incorrect loop detection outcomes.

Before performing ICP matching, it is necessary to process the key frames and candidate frames to eliminate their rotational sensitivity. To solve the issue of rotation sensitivity, researchers adopted a translation search method, which performs multiple translations on the scan context image of the reference frame, sequentially calculates the similarity with the scan context image of the current frame. By finding the translation with the highest similarity, the rotation angle between two frames is estimated. However, this brute-force search method inevitably increases the processing time of the system. Therefore, this paper utilizes prior information from VIS to reduce the number of searches. Acquiring a rough translation value through prior information, and then searching in a small range around this rough value, can shorten the search time.

Before the scan context, VIS uses a bag-of-words model to perform loop-closure detection first. We can use the prior information to roughly obtain rotational information and obtain the translation amount A (approximate value). $I_{1}$ is defined as the scan context of the current frame. $I_{2}$ is defined as the scan context of the reference frame. $I_{1}\left(A\right)$ represents the scan context after the current frame has been translated by A, and $I_{1}^{*}\left(A\right)$ is defined as the scan context that $I_{1}\left(A\right)$ has been translated by increment. $I_{1}^{*}\left(A\right)$ is performed:

(2) $$I_{1}^{*}\left(A\right)=I_{1}\left(A+\Delta h\right)$$

**Figure 8:**
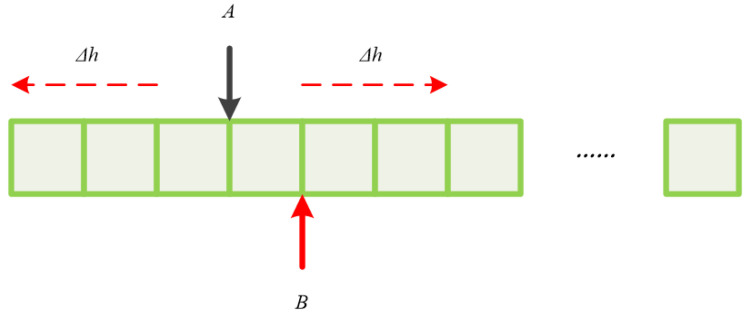
Schematic diagram of translation search method with prior information.

To obtain the precise translation amount B, it is only necessary to search in a small range near A. Figure 8 shows the schematic diagram of the translation search method with prior information. This method can use Formula (3) to calculate the similarity between the current frame $I_{1}^{*}\left(A\right)$ and the reference frame $I_{2}$.

(3) $$d\left(I_{1}^{*},I_{2}\right)=\frac{1}{N}\overset{N}{\underset{j=1}{\sum }}\left(1-\frac{c_{j}^{1}\cdot c_{j}^{2}}{\left\|c_{j}^{1}\|\|c_{j}^{2}\right\|}\right)$$

where N is defined as the number of columns in the ring-sector matrix, $c_{j}^{x}$ is defined as the column vector of the scan context, x is used to distinguish the category of frames, and j represents the column vector index of a certain frame. Each translation increment Δh yields a similarity measure, and the translation Δh with the smallest similarity measure is chosen; thus, the translation amount B is obtained according to Formula (4). Figure 7c is the scan context obtained after translation, and it can be seen that it is basically consistent with Figure 7a.

(4) B=A+Δh

## 3. Results and Discussion

The proposed methods have been experimented and validated on the datasets of KITTI, M2DGR, and the real-world scenario. In this section, we define metrics for evaluating the performance of SLAM systems and compare the effectiveness of the proposed method with the original LVI-SAM in terms of localization accuracy and mapping quality. Furthermore, the performance of our method and the original LVI-SAM has also been analyzed from both qualitative and quantitative perspectives.

### 3.1. KITTI Dataset Experiments

**Figure 9:**
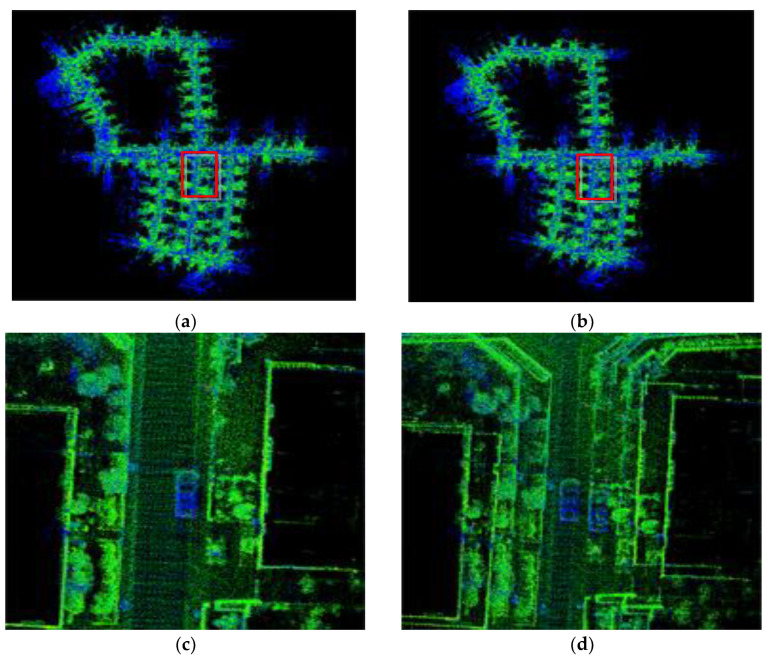
The mapping effects of different methods on KITTI sequence 05. (**a**) 3D mapping of the method proposed in this paper. (**b**) 3D mapping of LVI-SAM. (**c**) 3D map construction details of the method proposed in this paper. (**d**) 3D map construction details of LVI-SAM.

**Figure 10:**
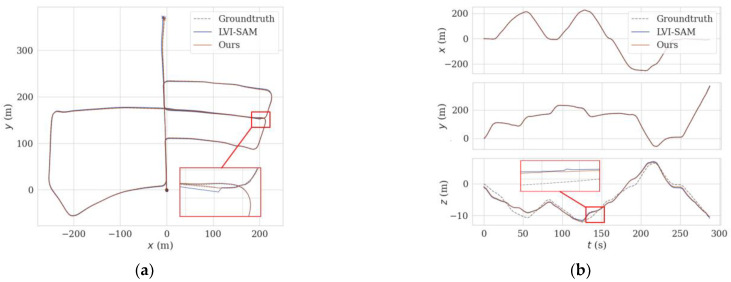
Comparison of trajectories using different methods on the KITTI sequence 05. (**a**) Comparison of trajectories on the x-y plane. (**b**) Comparison of trajectories in the x-, y-, and z-directions.

This experiment was conducted on the dataset of KITTI sequence 05. The localization accuracy and mapping effects of the proposed method and LVI-SAM were compared on this sequence. The results are shown in Figure 9 and Figure 10.

Figure 9a,b show the global maps constructed by the method proposed in this paper and LVI-SAM, and we analyzed the mapping effects using qualitative methods. On the dataset of KITTI sequence 05, due to the collection device passing through the position of the red solid-line box in the figure multiple times, loop detection occurred in the position. From the mapping details in Figure 9c,d, it can be seen that LVI-SAM experiences a phenomenon of overlapping loop-closure point clouds in this area, leading to a decrease in the quality of the mapping results. Our proposed method does not exhibit overlapping phenomena in this area, and the mapping effect is significantly better than LVI-SAM. Since the loop-closure detection module of our method uses the scan context module, it improves upon the loop-closure detection of the original LVI-SAM, enhancing the accuracy of loop-closure detection and enabling the acquisition of globally consistent maps.

Figure 10 shows the planar trajectory performance comparison of the method proposed in this paper and LVI-SAM. From the figure, it can be seen that at the position of the red solid-line box, there is a large-angle turn and a change in speed, which causes the trajectory of LVI-SAM to deviate, moving away from the true trajectory (shown as the dotted line) by a certain distance. Instead of the Shi–Tomasi algorithm of the original LVI-SAM, this paper utilizes SuperPoint + LK optical flow to extract and track feature points. In this degeneration scenario, it can effectively extract feature points, increase the success rate of matching, and then improve localization accuracy. Therefore, the trajectory of the method proposed in this paper is closer to the ground truth, and the localization accuracy is higher.

**Figure 11:**
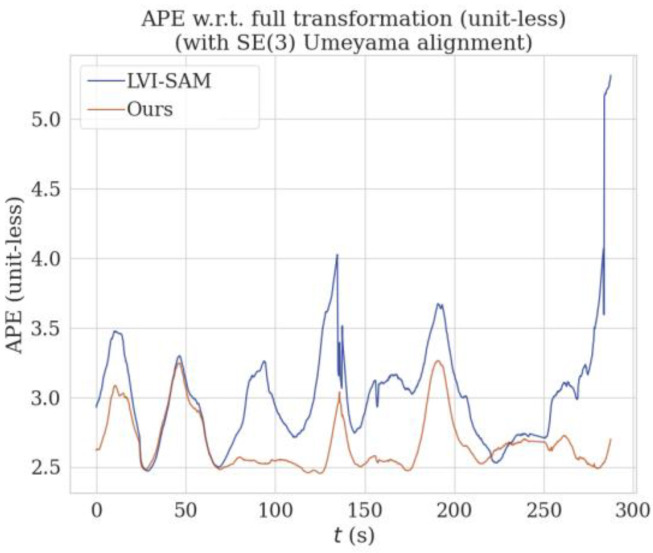
Comparison of APE at various time points on KITTI sequence 05 (/m).

The above results qualitatively analyze the localization and mapping effects of the proposed method and LVI-SAM on KITTI sequence 05. In order to verify the effectiveness and robustness of the proposed method for localization and mapping, the results use the EVO tool to conduct further quantitative analysis. Error curves of APE (absolute pose error) over time were plotted using the EVO tool for the proposed method and LVI-SAM. The results as shown in Figure 11 indicate that the APE of LVI-SAM is generally higher than the method proposed in this paper. Near t = 140 s, the APE curve of LVI-SAM shows significant fluctuations. As can be seen from the trajectory graph in Figure 10, there is a large-angle turning process at this time, leading to a degradation phenomenon, where the trajectory deviates from the true value, resulting in large errors. Our proposed method does not fluctuate very much here, indicating that SuperPoint feature point extraction is superior to the Shi–Tomasi algorithm in this degeneration scenario.

Finally, to quantify the APE curve, we recorded the maximum (MAX), minimum (MIN), standard deviation (STD), median, mean, and root mean square error (RMSE) [31] of the curve’s fluctuations over time into Table 4. STD and RMSE can better reflect the accuracy of the system. As indicated in Table 4, the proposed method outperforms LVI-SAM in terms of RMSE and STD, with errors reduced by 12% and 53%, respectively.

### 3.2. M2DGR Dataset Experiments

To verify the adaptability of the proposed method in different scenarios, we select the dataset of M2DGR sequence Street07 for experimentation and compare the localization accuracy of our method with that of LVI-SAM.

**Figure 12:**
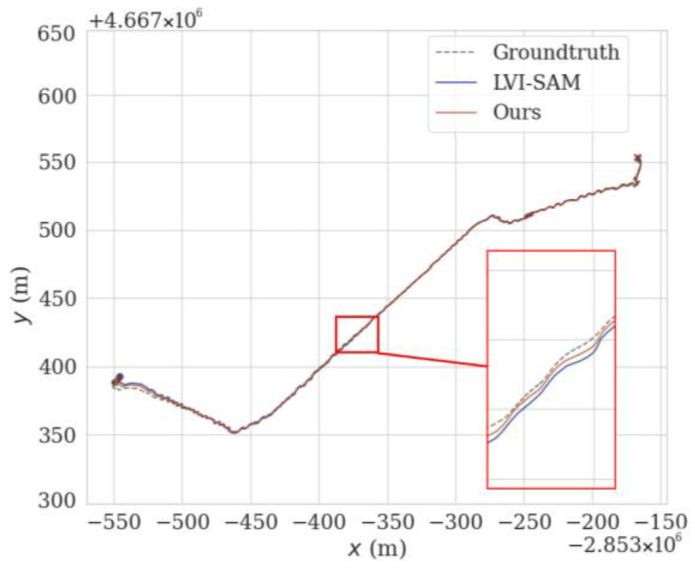
Comparison of trajectories using different methods on the M2DGR sequence Street07.

**Figure 13:**
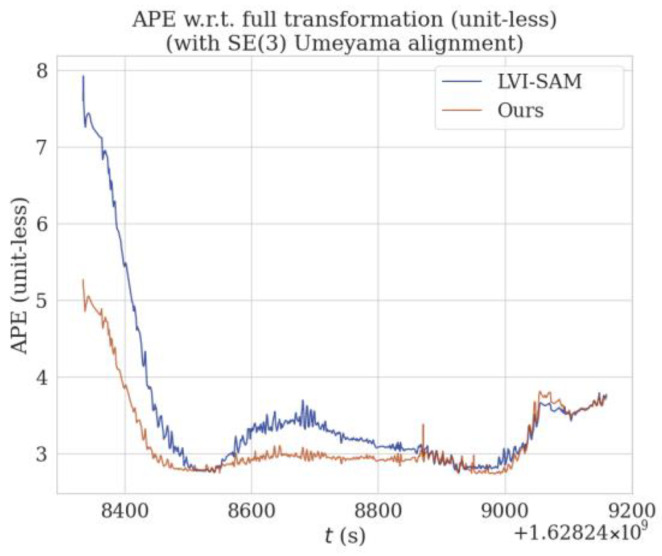
Comparison of APE at various time points on the M2DGR sequence Street07 (/m).

In the sequence Street07, there are many routes with winding and narrow areas, such as the red solid-line box in Figure 12, which lead to more drastic changes in angular velocity and cause increased distortion in the lidar data. Additionally, the data for this sequence were collected at night, with constantly changing lighting conditions; feature point detection is also quite difficult, as shown in Figure 2. Therefore, by incorporating SuperPoint into the VIS to extract feature points and descriptors in the method proposed in this paper, the accuracy of feature matching is improved, ensuring better localization accuracy and enhancing system performance. The red solid-line box in Figure 12 corresponds to the range where t is between 8600 and 8800 in Figure 13. This area is a winding and narrow strip. From the qualitative analysis of the figure, it can be seen that the APE of the method proposed in this paper is smaller than that of LVI-SAM, with less fluctuation. Similarly, the results use the EVO tool to calculate the APE of the proposed method and LVI-SAM for each parameter, which were recorded in Table 5, and by comparing the values of RMSE and STD in Table 5, we can conclude that the proposed method reduces the RMSE and STD by 11% and 50%, respectively, compared to LVI-SAM.

### 3.3. Real-World Scenario Dataset Experiments

**Figure 14:**
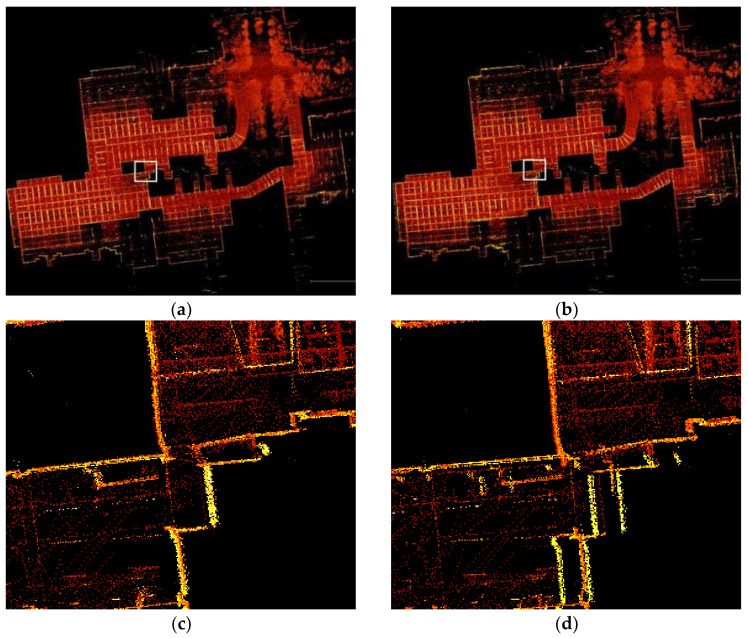
The mapping effects of different methods in real-world scenarios. (**a**) 3D mapping of the method proposed in this paper. (**b**) 3D mapping of LVI-SAM. (**c**) 3D map construction details of the method proposed in this paper. (**d**) 3D map construction details of LVI-SAM.

To further verify the effectiveness and robustness of the method proposed in this paper and to make the SLAM scenes more similar to the environment of animal farms, SLAM experiments were conducted in a simulated scenario using an experimental platform equipped with lidar, vision, and IMU (livestock inspection robot). Figure 14 shows the 3D mapping results of the proposed method and LVI-SAM in the real-world scenarios. It can be seen that both methods can produce high-quality 3D point cloud maps, but there are still significant differences in some mapping details. The areas marked by the white solid-line box in Figure 14a,b are the same region, and from the mapping details in Figure 14c,d, significant differences can be observed. The real-world scenario in this area is a white wall with a right-angle turn, and a ghosting blur phenomenon appears in Figure 14d, which is decided by the accuracy of the loop-closure detection. This area is the seventh segment of Figure 2, and when passing through this road section again, loop-closure detection occurs. Since our method incorporates the scan context loop-closure detection module, it improves the accuracy of loop-closure detection. It can be concluded that in the real-world scenario, the mapping effect is clearer than that of LVI-SAM.

**Figure 15:**
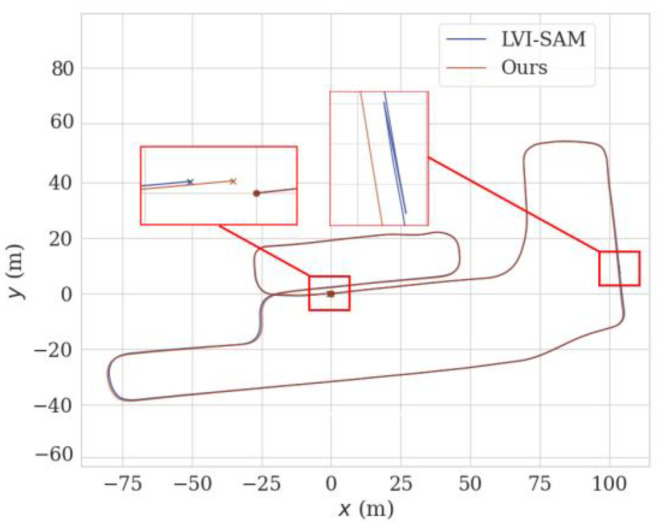
Comparison of trajectories using different methods in real-world scenarios.

**Figure 16:**
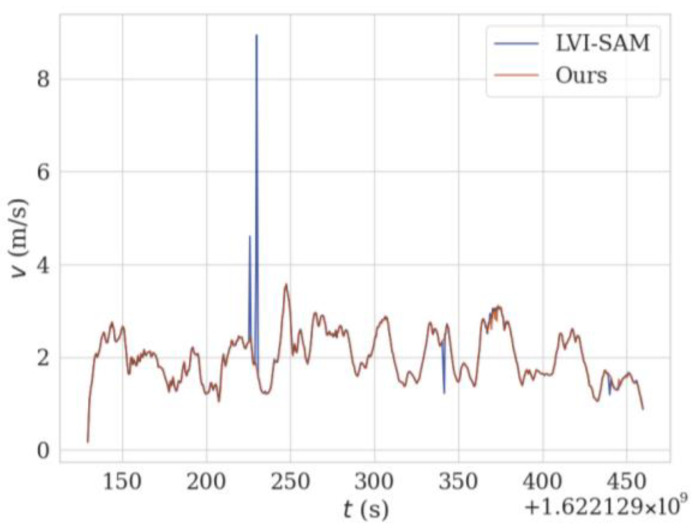
Movement speed using different methods at various times under real-world scenarios.

Figure 15 shows the trajectory graph of the proposed method and LVI-SAM running in a real-world scenario. Due to the unstable GPS signal reception, the ground truth cannot be obtained. Therefore, qualitative analysis can be conducted based on whether the starting and ending points of the livestock robot are consistent. Since the starting and ending points of the actual motion trajectory coincide, both the trajectory starting and ending points of the method proposed in this paper and LVI-SAM have shifted, with LVI-SAM having a greater trajectory deviation. Because the method proposed in this paper incorporates the scan context loop-closure detection and SuperPoint feature extraction module, which provides better robustness to complex scenarios, this results in the trajectory ending point being closer to the starting point and achieves higher localization accuracy. From Figure 15, it can also be observed that the trajectory of the LVI-SAM fluctuates during the fifth segment of Figure 2. This is due to a sudden jolt as the robot transitions from a downhill section to a flat area, causing a drastic change in the *z*-axis of IMU, resulting in trajectory fluctuation. From the blue pulse in Figure 16, it can also be seen that there was a significant deviation in the speed at this moment.

## 4. Conclusions

This paper proposes a multi-sensor fusion SLAM method based on the improved LVI-SAM, integrating data from lidar, vision, IMU, and other sensors for SLAM technology. We have made improvements to the original LVI-SAM in two aspects: visual feature point detection and loop-closure detection.

(1) Integrating the SuperPoint module into the VIS of LVI-SAM and replacing the Shi–Tomasi algorithm for feature point detection, which can better extract feature points in various complex visual scenarios. 

(2) Integrating the scan context module into the LIS of LVI-SAM for loop-closure detection, which can enhance the effectiveness and accuracy of loop-closure detection. 

The proposed method has been tested in complex environments such as KITTI, M2DGR, and real-world scenarios (simulating the operation scenes of animal farms). Experimental results indicate that the method proposed in this paper has higher localization accuracy and better robustness in some complex scenarios compared to LVI-SAM, enhancing the effectiveness and clarity of mapping in complex environments. 

However, the method also has some limitations, as the testing environment did not cover all real scenarios of animal farms. Future work could focus on further enriching the testing environment of real scenarios in animal farms, including the size and layout of the space, surface materials, lighting conditions, the influence of moving elements (such as animals), the presence of dust and particles, and the time scale, in order to cope with the complex working environment of livestock inspection robots.

## Figures and Tables

**Table 1:** The applicability of major existing sensors in SLAM.

Sensor	Advantage	Disadvantage	Degradation Scenarios
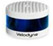 Lidar	High measurement accuracy Scale-free driftNo effect of light changes	Low scanning frequency Motion distortion	High-speed movement Narrow corridor Open environment
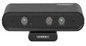 Camera	Rich texture information	Photosensitivity Scale uncertainty	Low texture Insufficient light
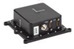 IMU	High output frequency High local precision	Cumulative error	Long-term error-free correction
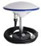 GPS	No cumulative error	Signal disturbance	Underground garages Tunnel

**Table 2:** Hardware parameters of the experimental platform.

Equipment	Version	Manufacturer, City, Country	Parameter
3D lidar	VLP-16	Velodyne, San Jose, CA, USA	16-line; 100 m measurement range; 300,000 points per second
2D lidar	MS200	ORADAR, Shenzhen, China	Single line; 12 m measurement range; 4500 points per second
Camera	AstraS	Orbbec, Shenzhen, China	640 × 480; 30 FPS
IMU	CMP10A	Yahboom, Shenzhen, China	Output frequency: 0.2–200 Hz; 10 axis
Processor	Jetson-Nano	NVIDIA, Santa Clara, CA, USA	4 Core A57; 472 GFLOPs
Motion chassis	TR500	HelloMaker, Shenzhen, China	Crawler-type; 0–1.2 m/s running speed

**Table 3:** Corresponding scenarios between experimental conditions and animal farms.

Number	Special Scenarios of Experimental Conditions	Scenarios of Animal Farms
1	Uphill	Road gradually ascending
2	Downhill	Road gradually descending
3	Exiting garage	Outdoor–Indoor switching
4	Entering garage	Outdoor–Indoor switching
5	Undulating jolting	Road surface uneven
6	Smooth straight movement	Normal inspection operation
7	Overlapping paths	Normal inspection operation
8	Sharp turns at large angles	Normal inspection operation

**Table 4:** Comparison of APE using different methods on KITTI sequence 05 (/m).

Method	MAX	MIN	STD	MEDIAN	MEAN	RMSE
LVI-SAM	5.31	2.47	0.41	3.01	3.03	3.06
Ours	3.27	2.46	0.19	2.59	2.66	2.67

**Table 5:** Comparison of APE using different methods on the M2DGR sequence Street07 (/m).

Method	MAX	MIN	STD	MEDIAN	MEAN	RMSE
LVI-SAM	7.92	2.75	0.84	3.14	3.36	3.47
Ours	5.27	2.74	0.42	2.94	3.06	3.09

## Data Availability

Data will be made available on request.

## Abbreviations

The following abbreviations are used in this manuscript:

SLAM	Simultaneous localization and mapping
IMU	Inertial measurement unit
ORB	Oriented FAST and rotated BRIEF
LIO	Lidar-inertial odometry
VIO	Visual-inertial odometry
LK	Lucas-Kanade
LIS	Lidar-inertial system
VIS	Visual-inertial system
ROS	Robot operating system
STD	Standard deviation
RMSE	Root mean square error
